# Delivering Multidomain Lifestyle Intervention Via Telehealth: Findings from a Brain Health Program in the United States

**DOI:** 10.1002/alz.095775

**Published:** 2025-01-09

**Authors:** Anitha Rao, Leyla Anderson, Travis Wilkes, Steven R Verdooner

**Affiliations:** ^1^ NeuroVision Imaging, Inc, Sacramento, CA USA

## Abstract

**Background:**

Multidomain lifestyle interventions have been shown to improve cognition and functional status in adults at elevated risk of developing dementia. The delivery of lifestyle interventions has primarily occurred during face‐to‐face clinical encounters. Unfortunately, this limits patients who live in rural and underserved areas, as well as patients who have mobility issues and transportation difficulties.

**Method:**

A lifestyle curriculum was developed and delivered using telehealth. The study enrolled patients who met the criteria of a Mini‐Mental State Examination score >18 and could participate in the study through telehealth. Referrals primarily came from neurology and primary care clinics. An electronic medical record system was utilized to document progress and improvement. All participants shared their wearable technology data. A BrainHealth Educator (BHE) delivered personalized digital content to participants, focusing on exercise, nutrition, and sleep. The content was tailored to each participant’s risk factors, APoE genotype, and communication preferences.

**Result:**

The study included 30 participants, with 23 females and 7 males ranging in age from 40 to over 70 years old. The enrollment and completion rates for exercise programs was 83%, for sleep programs was 83%, for nutrition programs was 58%, with an updated module currently being tested. The average duration of BHE telehealth visits was 30 minutes. There was high acceptance and satisfaction with communicating APOE test results via telehealth. 75% of participants actively used the app and 25% used the phone. 50% of patients logged in once per week. The study covered 9 geographic divisions across the US, achieving engagement across diverse practices, clinicians, and socioeconomic backgrounds, including neurologists, primary care physicians, and geriatricians. 75% of patients provided wearable data, with continuous wearable data submissions. The attrition rate was <10%.

**Conclusion:**

This study successfully demonstrates that lifestyle intervention delivered via telehealth by a BHE resulted in high patient engagement and satisfaction at nationwide scale. As cognitive health assessment and management rapidly develops, there is a need for lifestyle intervention programs that are structured to support patients at all stages and eliminate health disparities.

